# PKM2-Mediated Glycolytic Reprogramming in Thyroid Cancer: Mechanistic Insights and Therapeutic Potential

**DOI:** 10.3390/molecules31111811

**Published:** 2026-05-25

**Authors:** Shenshen Li, Wei Liu, Jiaojiao Zheng, Lingyu Ren, Changhao Zhou, Qiao Wu, Zhilong Ai

**Affiliations:** 1 Department of Surgery (Thyroid & Breast), Zhongshan Hospital, Fudan University, Shanghai 200030, China; 2 School of Basic Medical Sciences, Fudan University, Shanghai 200433, China

**Keywords:** PKM2, thyroid cancer, metabolic reprogramming

## Abstract

Thyroid cancer (TC) is an endocrine malignant tumor with the fastest-growing incidence worldwide. It has complex pathological types and significant heterogeneity, with great differences in clinical prognosis among different subtypes. Among them, aggressive subtypes, such as radioiodine-refractory (RAI-R) TC and anaplastic thyroid cancer (ATC), have become a major challenge in current clinical diagnosis and treatment, due to limited treatment options and high risks of recurrence and metastasis. Tumor metabolic reprogramming is one of the characteristics of cancer, among which the Warburg effect plays a driving role. As a rate-limiting enzyme in the glycolytic pathway, pyruvate kinase M2 (PKM2), with its unique functional plasticity, has become a linchpin of glycolytic metabolism and malignant phenotypes of tumor cells. This article will systematically review the functional regulatory mechanisms of PKM2, its specific role in TC, and explore the targeted therapeutic strategies and research prospects of TC with PKM2, providing a new theoretical basis and potential plans for the clinical diagnosis and treatment.

## 1. Introduction

Thyroid cancer (TC), the most common endocrine malignancy, continues to exhibit a rising global incidence, posing a significant public health challenge [1,2]. Globally, the incidence of TC is showing a continuous upward trend. According to the statistics from the International Agency for Research on Cancer (IARC) of the World Health Organization, there were 586,000 new cases of TC and about 44,000 deaths worldwide in 2020, and the incidence rate in women was significantly higher than that in men, with a male-to-female incidence ratio of about 1:3, and the age of onset was largely in young and middle-aged groups [3,4,5]. In China, the incidence of TC is also rising rapidly. Data released by the National Cancer Center of China in 2022 showed that TC has ranked among the top three malignant tumors in Chinese women, and the incidence is showing a clear younger trend, posing a severe challenge to public health [6].

According to histological phenotypes and differentiation degree, thyroid cancer is mainly divided into four categories: differentiated thyroid cancer (DTC), anaplastic thyroid cancer (ATC), medullary thyroid cancer (MTC), and other rare subtypes [7]. Among them, DTC accounts for the highest proportion, approximately 90% of all TC cases, and is further divided into papillary thyroid cancer (PTC) and follicular thyroid cancer (FTC). Such tumors have low malignant degree and good prognosis after early surgery combined with radioactive iodine (RAI) therapy, but some patients still experience recurrence, metastasis, or radioiodine resistance [8]. Although ATC accounts for less than 5% of cases, it has an extremely high malignant degree, strong invasiveness, poor sensitivity to radiotherapy and chemotherapy, and the median survival time is usually less than 1 year [9]. MTC originates from parafollicular C cells of the thyroid gland, and its occurrence is closely related to RET proto-oncogene mutations, with both hereditary and sporadic hallmarks, and its prognosis is between DTC and ATC [6,10].

Standard treatment approaches for DTC include surgery, thyroid hormone suppression therapy, and RAI therapy [11,12]. However, approximately 5% to 15% of DTC patients develop RAI-refractory (RAI-R) disease, wherein tumor cells lose the capacity to uptake iodine, rendering RAI therapy ineffective [13]. One study reported that up to 60% of patients with aggressive metastatic DTC develop resistance to RAI treatment [14]. The emergence of RAI resistance significantly worsens patient prognosis and elevates the risk of distant metastasis [15]. For RAI-R DTC patients, treatment options become markedly of disease recurrence and limited. Multi-kinase inhibitors (MKIs), such as sorafenib and lenvatinib, have demonstrably improved progression-free survival in these patients, yet their objective response rates (ORR) typically range from 12% to 27%, often accompanied by significant toxicities that impair quality of life [16,17].

The occurrence and development of TC is a complex process involving multiple gene mutations and aberrant activation of signaling pathways. The identification of driving genes provides an important basis for understanding their pathogenesis and precise treatment. The BRAF V600E mutation is the most common gene mutation in TC, with an incidence rate of 40–66% in PTC and about 24% in ATC [18,19]. This mutation can make the BRAF protein continuously activated, independent of upstream RAS signals, break the balance of cell growth by phosphorylating the downstream MEK-ERK pathway, and promote metabolic reprogramming, which is closely related to tumor TNM stage, lymph node metastasis, and poor prognosis [20,21]. RAS gene mutations (including NRAS, HRAS, and KRAS) are more common in FTC, MTC, and some PTC, with an incidence rate of about 10–20%, regulating tumor cell growth and survival by activating PI3K-AKT and MAPK pathways [22]. Additionally, RET/PTC rearrangement, TERT promoter mutation, P53 mutation, etc., are also cornerstones of different subtypes of TC. Among them, the TERT promoter mutation is more common in advanced DTC and ATC, indicating increased tumor invasiveness and poor prognosis [23].

## 2. Metabolic Reprogramming, Warburg Effect and PKM2

Tumor metabolic reprogramming is widely recognized as one of the central characteristics of malignant tumors, among which the Warburg effect (aerobic glycolysis) is the most typical manifestation [24]. This phenomenon describes how tumor cells preferentially choose the glycolytic pathway to convert glucose into lactic acid even under aerobic conditions, rather than performing efficient energy metabolism through oxidative phosphorylation (OXPHOS). This phenomenon is significantly different from the metabolic mode of normal cells [25]. The Warburg effect is of great significance for the malignant proliferation and microenvironmental adaptation of tumor cells. Its value lies not only in rapidly providing energy for tumor cells but also, more importantly, in shunting glucose metabolic intermediates (such as triose phosphate, pyruvate, ribose phosphate, etc.) to biosynthetic pathways such as nucleotides, amino acids, and lipids through regulating glycolytic flux, providing sufficient material basis for the rapid proliferation of tumor cells [24,26,27]. At the same time, the Warburg effect helps tumor cells adapt to harsh tumor microenvironments (TME) such as hypoxia and nutrient deficiency by maintaining intracellular reactive oxygen species (ROS) homeostasis and regulating fundamental signaling pathways [28].

Pyruvate kinase (PK) is a principal terminal enzyme in the glycolytic pathway, which can catalyze the conversion of phosphoenolpyruvate (PEP) to pyruvate and generate ATP. Its activity directly regulates the rate and direction of glycolytic flux [29]. According to the specificity of gene coding and tissue distribution, the pyruvate kinase family is mainly divided into four subtypes: liver-type (PKL), red blood cell-type (PKR), muscle-type 1 (PKM1), and muscle-type 2 (PKM2) [30]. Among them, PKL is chiefly expressed in tissues such as the liver, kidneys, and small intestine, participating in the regulation of glucose metabolism homeostasis; PKR is specifically expressed in red blood cells and renal medulla, ensuring the energy supply of red blood cells; both PKM1 and PKM2 are encoded by the PKM gene and produced through alternative splicing, and their tissue distribution and functional characteristics have significant differences [31].

**Figure 1:**
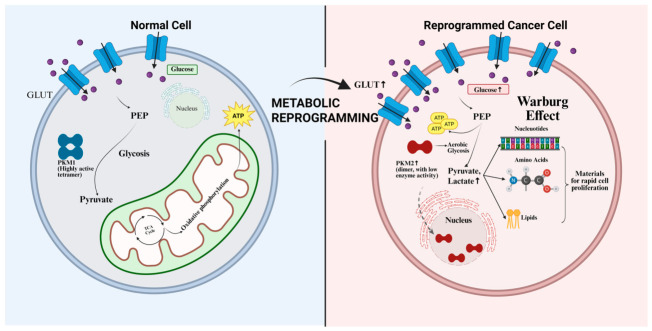
Metabolic reprogramming, Warburg effect, and the role of pyruvate kinase M2 (PKM2) in thyroid cancer. (**Left**) Normal cellular metabolism: Under normal physiological conditions, glucose is converted into pyruvate through glycolysis by the highly active tetramer PKM1, which then enters mitochondria for the tricarboxylic acid cycle (TCA cycle) and oxidative phosphorylation, producing a large amount of ATP. (**Right**) Metabolic reprogramming and Warburg effect in thyroid cancer cells: Cancer cells exhibit metabolic reprogramming, prioritizing glycolysis even under aerobic conditions, converting most glucose to lactate and excreting it from the cell, a phenomenon known as the “Warburg effect”. In thyroid cancer cells, abundant pyruvate kinase M2 (PKM2) typically exists in a low activity dimer form, leading to the accumulation of glycolytic intermediates in the cytoplasm. These accumulated intermediate products are diverted to the biosynthetic pathway, providing large molecular precursors, such as nucleotides, amino acids, and lipids, for the rapid proliferation of cancer cells. PKM2 can simultaneously relocate to the cell nucleus, which will be discussed later. Created in Biorender. Shenshen Li. (2026) http://BioRender.com/.

PKM1 is mostly found in metabolically active normal tissues such as myocardium, skeletal muscle, and brain tissue. Because it contains exon 9 of the PKM gene, it can stably exist in the form of a high-activity tetramer, with strong enzyme activity and is not easily regulated by external signals, which can efficiently catalyze glycolytic reactions and ensure cell energy supply. In contrast, PKM2 contains exon 10 of the PKM gene, which is mostly enriched in embryonic tissues, stem cells, and malignant tumor cells, with high structural and functional plasticity, and can dynamically switch between tetramer and dimer forms: the tetramer form of PKM2 has high enzyme activity, promoting the complete progress of glycolysis; the dimer form has significantly reduced enzyme activity, which can lead to the accumulation of glycolytic intermediates and provide raw materials for biosynthetic pathways [32,33,34]. As shown in Figure 1, cancer cells preferentially undergo aerobic glycolysis through metabolic reprogramming, particularly the Warburg effect, to support their rapid proliferation. This process involves the dimer form of PKM2, which features low enzyme activity compared with the tetramer form of PKM1. Therefore, PKM2 plays a universal and critical role in cancer metabolic reprogramming. As a rate-limiting enzyme in the glycolytic pathway, PKM2 promotes aerobic glycolysis and biosynthetic processes by modulating metabolic flux in tumor cells to meet the demands of rapid proliferation [35,36,37].

## 3. Regulation and Modification of PKM2

In TC cells, BRAF V600E mutation can induce PKM2 expression by activating the downstream MAPK signaling pathway [38,39]. Even under normoxic conditions, BRAF V600E-mediated MAPK overactivation can upregulate PKM2 expression through the stabilization of c-Myc and HIF-1α [40,41]. This upregulation promotes PKM2 to form its low-activity dimeric form, thereby enhancing aerobic glycolysis, which provides biosynthetic precursors for rapid tumor growth [33]. Besides the MAPK/ERK signaling pathway, the BRAF V600E mutation targets the PI3K pathway as well [42]. This is also a possible axis that connects BRAF V600E mutation and PKM2, because a previous study shows AKT kinase, as a key component of the PI3K/Akt signaling pathway, can phosphorylate the Ser37 site of PKM2, thereby affecting the cellular localization and function of PKM2 [43].

RAS mutations are a common driving factor for thyroid follicular carcinoma [44]. Its carcinogenic core lies in the continuous activation of downstream MAPK and PI3K-AKT signaling pathways; therefore, it has similarities with BRAF V600E in regulating PKM2 [45]. The incidence of TERT promoter mutations is increased in poorly differentiated cancer, undifferentiated cancer, and invasive papillary carcinoma, and is closely related to poor prognosis [46]. Its direct function is to enhance telomerase activity, maintain telomere length, and promote cell immortality [47]. Although not directly encoding signal proteins, TERT mutations often coexist with BRAF or RAS mutations, and this synergistic effect greatly enhances the invasiveness of tumors [48]. From a metabolic perspective, upregulation of telomerase activity is associated with cellular metabolic reprogramming. There are studies suggesting that TERT may affect the activity of transcription factors, such as c-Myc in a non-telomerase-dependent manner, and c-Myc is a key transcriptional regulator of PKM2 expression [49,50].

P53 mutations are extremely common in progressive TC, especially ATC [51]. The wild-type p53 protein is an important negative regulator of cellular metabolism, which can inhibit glycolysis and promote OXPHOS [52]. p53 can inhibit glycolysis flux by inducing the expression of TIGAR (p53-induced glycolysis and apoptosis regulatory factor). When p53 mutates and becomes inactive, this braking effect on glycolysis is released, which may lead to a general upregulation of glycolytic enzymes, including PKM2, accelerating the Warburg effect [53].

**Figure 2:**
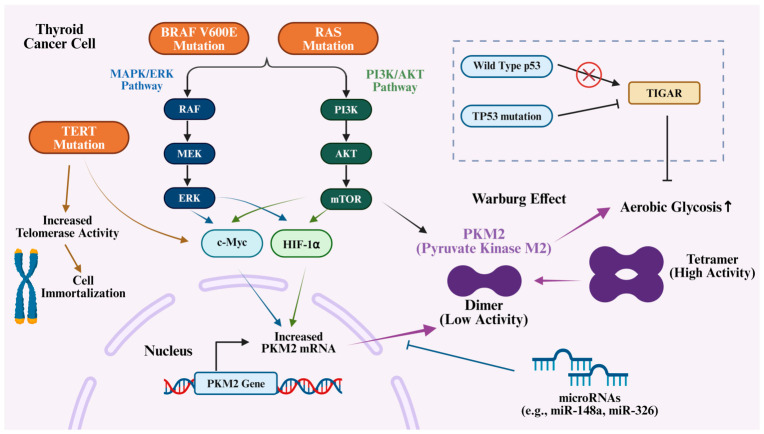
BRAF V600E and RAS mutations activate the MAPK/ERK (RAF→MEK→ERK) and PI3K/AKT (PI3K→AKT→mTOR) signaling cascades, respectively. Both pathways converge on the transcription factors c-Myc and HIF-1α, which translocate into the nucleus and promote the transcriptional upregulation of the PKM2 gene, leading to increased PKM2 mRNA levels. Additionally, the BRAF V600E mutation enhances telomerase activity, contributing to cell immortalization. The interconversion between the tetramer and dimer forms is a key regulatory switch in tumor metabolic reprogramming. Post-transcriptional regulation by microRNAs (e.g., miR-148a, miR-326) modulates PKM2 expression by targeting PKM2 mRNA. In the context of tumor suppressor loss, wild-type p53 normally activates TIGAR (TP53-induced glycolysis and apoptosis regulator), which suppresses aerobic glycolysis; however, p53 mutations abrogate this regulatory axis, resulting in the loss of TIGAR-mediated glycolytic inhibition and further enhancement of the Warburg effect. Created in Biorender. Shenshen Li. (2026) http://BioRender.com/.

Beyond genetic mutations, epigenetic regulation via miRNAs adds another layer of control over PKM2 expression. Those pathways uniquely modify PKM2 in TC are summarized in Figure 2. Other PKM2 regulation mechanisms are listed in Table 1, and these provide potential research directions for future experimental validation.

**Figure 3:**
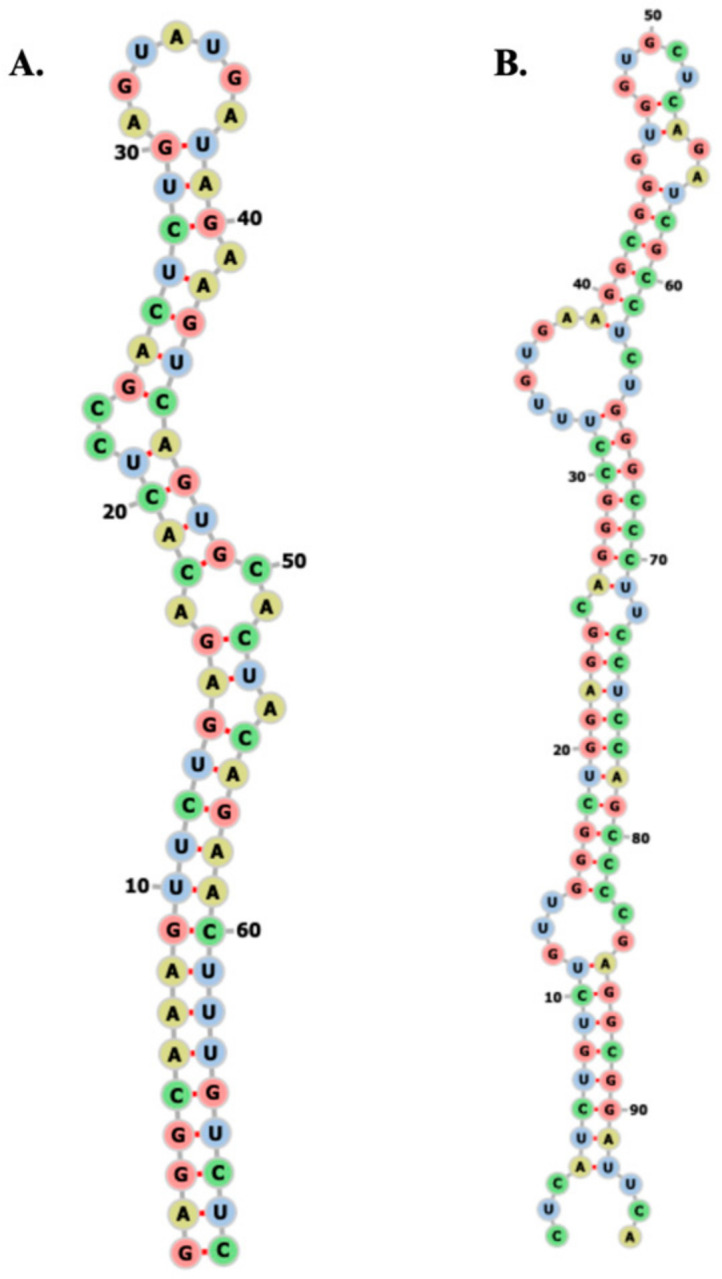
(**A**) pre-miR-148a; (**B**) pre-miR-326. They are obtained from miRBase (http://www.mirbase.org) (accessed on 8 April 2026) and visualized using RNAfold.

**Figure 4:**
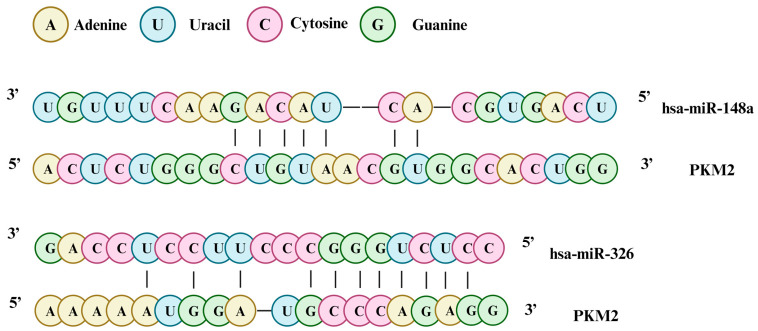
Predicted putative miR-148a and miR326-binding sites in the 3′-untranslated region (UTR) of PKM2. Created in Biorender. Shenshen Li. (2026) http://BioRender.com/.

As a class of non-coding small RNAs, miRNAs can induce transcriptional or translational inhibition by binding to the 3′ untranslated region (3′UTR) of target gene mRNAs, participating in the post-transcriptional regulation of PKM2 and enriching the regulatory dimensions of PKM2 [80,81]. As a tumor-suppressive miRNA, miR-122 is downregulated in TC. It can directly target the 3′UTR of PKM2 to inhibit PKM2 expression, thereby reversing the Warburg effect and inhibiting tumor cell proliferation [55]; while miR-326 synergistically inhibits the invasion and metastasis of thyroid cancer cells by targeting PKM2 and downstream signaling pathway molecules. In addition, miR-148a, miR-326, etc., have also been confirmed to regulate PKM2 expression, and anomalous expression of these miRNAs in TC further disrupts the expression balance of PKM2 and promotes TC progression [54]. Figure 3 demonstrates the secondary structures of pre-miR-148a and pre-miR-326. They are obtained from miRBase (http://www.mirbase.org) (accessed on 8th April, 2026) and visualized using RNAfold. Figure 4 shows predicted putative miR-148a and miR-326-binding sites in the 3′UTR of PKM2.

Post-translational modification (PTM) is the central way to regulate PKM2 enzyme activity, subcellular localization, and protein–protein interactions, mainly including phosphorylation, acetylation, oxidation, and other modification types. Different modification methods can dynamically regulate its function by changing the spatial conformation of PKM2. These modifications are abnormally activated in TC, further enhancing the pro-cancer function of PKM2 [82,83].

Phosphorylation modification is the most common PTM method of PKM2. A variety of kinases can regulate its enzyme activity, subcellular localization, and non-enzymatic functions by phosphorylating different amino acid sites of PKM2, among which FGFR1, AKT, and ERK are focal regulatory kinases [58]. FGFR1 can specifically phosphorylate the Tyr105 site of PKM2, inducing PKM2 to dissociate from the high-activity tetramer into the low-activity dimer, inhibiting its enzyme activity, and promoting the nuclear translocation of the dimer form of PKM2 to exert the function of a transcriptional coactivator. The activation of the FGFR1-PKM2 signaling axis in TC can synergize with the BRAF V600E mutation to promote tumor metabolic reprogramming and invasion and metastasis [59].

AKT can enhance the interaction between PKM2 and HIF-1α by phosphorylating the Ser37 site of PKM2, promote PKM2 nuclear translocation, and inhibit its enzyme activity, enhancing the Warburg effect [60], while ERK, as a pivotal downstream kinase of the MAPK pathway, can phosphorylate the Thr365 site of PKM2, regulate its binding ability to β-catenin, and promote the expression of proliferation-related genes [58]. In TC, BRAF V600E and RAS mutations can activate AKT and ERK kinases, leading to dysregulated phosphorylation modification of PKM2, forming a “driver gene-kinase-PKM2” regulatory pathway, which synergistically promotes tumor progression [57]. Phosphorylation modifications at different sites can precisely regulate the functional state of PKM2 through synergistic or antagonistic effects, providing multi-dimensional targets for the targeted therapy of TC.

Tripartite motif-containing protein 35 (TRIM35) is a member of the RBCC family, which has a highly conserved sequence: a RING domain followed by one or two B-Box domains, and then a coiled-coil domain [84]. In hepatocellular carcinoma, the coiled-coil domain mediates the reduction in the Warburg effect and cancer cell proliferation. In addition, TRIM35 also inhibits the tumorigenicity of HCC cells by blocking PKM2 Tyr105 phosphorylation [56,85].

Crotonylation of PKM2 can upregulate and promote its nuclear translocation, subsequently impacting metabolic reprogramming and fostering phenotypic switching in vascular smooth muscle cells [61]. Similar crotonylation in tumors may also drive tumor progression by affecting PKM2’s subcellular localization and function [86].

Succinylation modification maintains the low-activity state by changing the conformation of PKM2 and inhibiting its tetramer formation [62]. Studies have shown that SIRT5 (a desuccinylase) removes succinylation modifications on specific lysine residues of PKM2, thereby promoting tetramer formation. Conversely, accumulation of succinylation disrupts the tetramer interface, maintaining PKM2 in a low-activity dimeric/monomeric state [63].

PKM2 is also modified by O-linked β-N-acetylglucosamine (O-GlcNAc). The enzyme OGT (O-GlcNAc transferase) attaches an O-GlcNAc group to PKM2. Through mass spectrometry, the researchers identified Thr405 as the specific site of this modification. Thr405 is located at the critical interface where PKM2 monomers bind to form a tetramer. The addition of the O-GlcNAc group creates steric hindrance, destabilizing the highly active tetramer and shifting PKM2 into the low-activity dimeric or monomeric state [64].

Acetylation modification predominantly regulates the balance between dimer and tetramer of PKM2 and its enzyme activity by changing the spatial conformation of PKM2, among which p300 is the core acetyltransferase regulating PKM2 acetylation [69,74]. p300 can specifically acetylate sites such as Lys305 and Lys433 of PKM2, inducing PKM2 to dissociate from the high-activity tetramer into the low-activity dimer, inhibiting its glycolytic enzyme activity, and promoting the nuclear translocation of the dimer to enhance its interaction with transcription factors and exert the function of a transcriptional coactivator [68]. However, the deacetylase SIRT1 can reverse the acetylation at Lys305 site, forming a dynamic balance of acetylation modification. This balance is disrupted in non-small cell lung cancer (NSCLC), becoming a crucial regulatory mechanism of metabolic reprogramming [65].

Under the oxidative stress microenvironment, PKM2 can undergo oxidative modification, among which Cys358 is a cardinal oxidative site [87]. Oxidative modification can directly regulate the enzyme activity and function of PKM2 [70]. Due to the active metabolism and hypoxic microenvironment of cancer cells, the intracellular level of ROS is significantly increased, which can induce oxidative modification of the Cys358 site of PKM2, forming disulfide bonds, resulting in complete loss of PKM2 enzyme activity, and promoting its nuclear translocation to interact with transcription factors such as HIF-1α and p53 to regulate downstream gene expression [88]. Oxidatively modified PKM2 can help TC cells resist ROS-induced apoptosis by activating antioxidant stress-related genes, and enhance the Warburg effect to adapt to the oxidative stress microenvironment [71].

Fructose-1,6-bisphosphate (FBP) is a salient allosteric activator of PKM2, which can specifically bind to the allosteric site of PKM2, inducing its transformation from the low-activity dimer to the high-activity tetramer, significantly enhancing its glycolytic enzyme activity, promoting the complete progress of glycolysis, and reducing the accumulation of intermediates [75,77]. In the early stage of TC, the glucose concentration in the TME is relatively sufficient, and the FBP level is high, which can activate the enzyme activity of PKM2 to provide energy for tumor cells; while in the advanced stage of tumor, local hypoxia and nutrient deficiency lead to a decrease in FBP concentration, and PKM2 returns to the low-activity dimer form, initiating metabolic reprogramming to provide raw materials for biosynthesis [74]. In addition, the allosteric activation of PKM2 by FBP can also inhibit its nuclear translocation function, reducing the pro-cancer effect mediated by non-enzymatic activity [72].

Serine can bind to the active center of PKM2, competitively inhibit its enzyme activity, and promote its nuclear translocation to exert the function of a transcriptional coactivator [78]. Serine regulates PKM2 enzymatic activity by binding to its amino acid binding pocket. Chaneton et al. resolved the three-dimensional structure of the PKM2–L-serine complex at a resolution of 2.30 Å using X-ray crystallography, providing direct evidence for the molecular details of the serine–PKM2 interaction. Structural analysis revealed that serine binding to PKM2 primarily relies on a key residue in the AA binding pocket—His464 is essential for serine binding and activation of PKM2; the H464A mutant completely loses both serine binding ability and the corresponding activation effect [79].

## 4. Function of PKM2 in Cancer

PKM2 promotes aerobic glycolysis, diverting metabolic intermediates into biosynthetic pathways to meet the macromolecular demands for nucleotides, amino acids, and lipids required for rapid tumor cell proliferation. TC cells exhibit metabolic reprogramming characteristics such as mitochondrial dysfunction, activated glycolysis, lipid metabolism imbalance, and glutamine dependence [89,90]. For example, recent research indicates that transketolase (TKT) synergizes with PKM2 to promote renal cell carcinoma progression by enhancing glycolysis [91].

**Figure 5:**
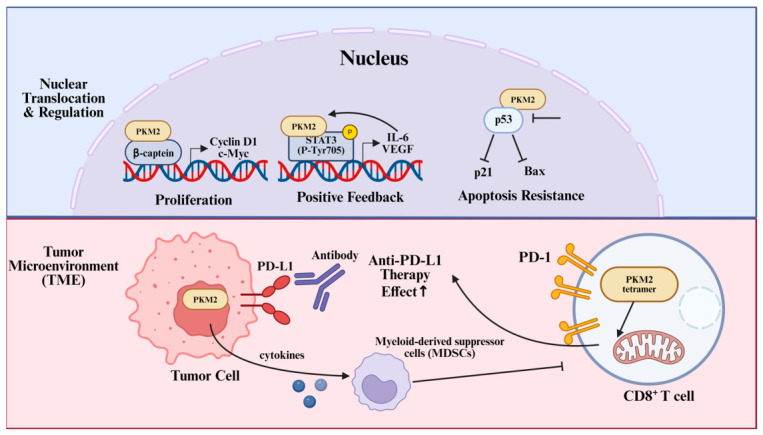
Nuclear PKM2 drives tumor proliferation and apoptosis resistance via β-captein, STAT3, and downregulate p53 transcription. In the TME, PKM2 promotes cytokine expression and MDSCs accumulation, suppressing T cells. PKM2 tetramer in CD8^+^ T cells can enhance the effect of anti-PD-L1 therapy. Created in Biorender. Shenshen Li. (2026) http://BioRender.com/.

The non-metabolic functions of PKM2 also play a critical role in TC progression [92,93]. As shown in Figure 5, PKM2 can translocate into the cell nucleus, acting as a transcriptional coactivator involved in regulating gene expression, thereby influencing tumor cell proliferation, invasion, and metastasis. The interaction with β-catenin is one of the dominant mechanisms by which PKM2 regulates tumor proliferation. Nuclear PKM2 can bind to β-catenin, promote its translocation into the nucleus, and enhance the binding ability of β-catenin to T-cell factor/lymphoid enhancer factor (TCF/LEF) family transcription factors, upregulating the expression of proliferation-related genes, such as Cyclin D1 and c-Myc, and promoting the cell cycle progression of TC cells [94,95]. In addition, PKM2 can enhance the stability of β-catenin by phosphorylating it, inhibit its ubiquitin-dependent degradation, and further strengthen the pro-cancer signal mediated by β-catenin [90,96].

As a primary regulatory factor of inflammation and tumor progression, STAT3 is disorderedly activated in PTC and can interact with nuclear PKM2 to form a positive feedback regulatory loop [97,98]. PKM2 can activate the transcriptional function of STAT3 by phosphorylating the Tyr705 site of STAT3, upregulating the expression of genes such as IL-6 and VEGF; while activated STAT3 can in turn promote PKM gene transcription, enhance PKM2 expression, and synergistically promote the proliferation, invasion, and immune suppression of TC cells [99,100].

Existing clinical studies and basic experiments have confirmed that PKM2 shows abnormally high expression in TC tissues, and its expression level is significantly correlated with tumor invasiveness and clinical prognosis, having potential value as a prognostic biomarker for TC [101]. In clinical sample studies, detection by immunohistochemistry, real-time fluorescence quantitative PCR, Western blotting, and other technologies found that the positive expression rate and expression level of PKM2 in TC tissues were significantly higher than those in adjacent normal thyroid tissues, and there were obvious subtype-specific differences—in ATC, highly invasive PTC (such as diffuse sclerosing type, tall cell type), and MTC, the expression level of PKM2 was significantly increased, while in low-malignancy classic PTC and FTC, its expression level was relatively low, suggesting that PKM2 expression is positively correlated with the malignant degree of TC [102].

Analysis of clinicopathological correlation showed that high PKM2 expression was closely related to adverse clinical characteristics of TC patients. Specifically, the expression level of PKM2 was positively correlated with tumor size. In TC tissues with a maximum diameter >2cm, the positive expression rate of PKM2 was significantly higher than that in small tumor tissues; Meanwhile, patients with high PKM2 expression were more likely to have cervical lymph node metastasis, distant metastasis (such as lung metastasis, bone metastasis), and a later TNM stage (higher proportion of stage III–IV) [101]. Survival analysis results further confirmed that the disease-free survival (DFS) and overall survival (OS) of patients in the high PKM2 expression group were significantly shorter than those in the low expression group. Multivariate COX regression analysis showed that high PKM2 expression was an independent risk factor for poor prognosis of TC patients [103].

Evidence from cell and animal experiments further verified the pro-cancer effect of PKM2 in TC. In vitro experiments, high expression of PKM2 was detected in TC cell lines (papillary cancer TPC-1, B-CPAP cells, anaplastic cancer 8505C, SW1736 cells, medullary cancer TT, MZ-CRC-1 cells), and after knocking down PKM2 expression, the proliferation activity and colony formation ability of cancer cells were significantly decreased, while overexpression of PKM2 could enhance the malignant phenotype of cancer cells [99,102]. In vivo xenograft model studies showed that when PKM2-silenced TPC-1 cells were subcutaneously injected into nude mice, the growth rate of xenografts was significantly slowed down, and the tumor volume and weight were significantly smaller than those in the control group [104]; In the meantime, in the lung metastasis model, PKM2 knockdown could significantly reduce the number of lung metastases in nude mice, confirming that PKM2 plays an irreplaceable role in the in vivo growth and metastasis of TC [105].

Besides TC, PKM2 is widely investigated in other cancers. The interaction between PKM2 and p53 has a bidirectional regulatory characteristic, participating in the precise regulation of the cell cycle and apoptosis [106]. Under stress conditions, such as oxidative stress and DNA damage, nuclear PKM2 can bind to p53, inhibit the transcriptional activity of p53, downregulate the expression of apoptosis-related genes, such as p21 and Bax, and help cancer cells resist stress-induced apoptosis [107], while in low-malignancy TC cells, p53 can in turn inhibit the nuclear translocation function of PKM2, maintain its cytoplasmic enzyme activity, and avoid metabolic reprogramming. Meanwhile, p53 mutation leads to loss of function, breaking the balance of interaction with PKM2 via the mTOR signaling pathway, and further enhancing the pro-cancer function of PKM2 [108].

As shown in Figure 5, PKM2’s role in the TME also includes the regulation of immune cells, particularly macrophage polarization [109]. Macrophages can polarize into two functionally distinct phenotypes based on microenvironmental signals: M1, which exhibits pro-inflammatory and anti-tumor effects, and M2, which promotes tissue repair, angiogenesis, and tumor progression [110,111]. PKM2 can regulate M1/M2 polarization of macrophages and cytokine secretion, thus affecting inflammation and tumor progression [112]. While lactic acid, which increases during the Warburg effect, has the same effect on TAMs [113]. It can also accelerate the formation of cancer-associated fibroblasts (CAFs) in pancreatic cancer [114]. PKM2 dimer promotes the release of cytokines by tumor cells, thereby recruiting myeloid-derived suppressor cells (MDSCs). These cytokines bind to the surface receptors of MDSCs and activate related signaling pathways. This process subsequently inhibits the activity of T cells, thereby affecting tumor development [115,116,117]. On the opposite, PKM2 tetramer can enhance mitochondrial biogenesis and effector function of CD8^+^ T cells. This phenomenon can improve the efficacy of PD-L1 therapy [118].

## 5. PKM2 in Different Subtypes of TC

DTC is primarily divided into PTC and FTC, which exhibit significant differences in molecular characteristics and clinical behavior [39,119].

PTC is the most common subtype of TC, generally with a good prognosis but a nearly 40% of central lymph node metastasis [120]. One study found that the expression of glycolytic enzymes, including PKM2, is linked to aggressive features of PTC, and this association differs in patients with or without chronic lymphocytic thyroiditis (CLT) [121]. Specifically, studies have found that PKM2 mRNA expression may serve as a biomarker for lymph node metastasis in PTC [122]. Single-cell transcriptomic analysis has revealed heterogeneity in tumor and immune cells during PTC lymph node metastasis, as well as ITGA2-mediated metabolic reprogramming and immune crosstalk [123,124,125].

FTC is the second most common type of TC, characterized by lower aggressiveness but sometimes developing distant metastases. Unlike PTC, FTC typically metastasizes hematogenously rather than via lymph nodes [126]. While PKM2 also promotes proliferation and metastasis in other tumors, direct research on the specific role of PKM2 in FTC is limited.

While research on the role of PKM2 in ATC remains limited, evidence from other malignancies has established a significant correlation between nuclear PKM2 accumulation and tumor proliferation [127]. Therefore, future studies should explore whether nuclear PKM2 levels vary across different thyroid cancer subtypes and further investigate their potential association with cell proliferation, migration, and metastatic capacity.

In RAI-R TC, except that PKM2 dimer has a negative effect on SLC5A5 and thyroglobulin through histone H3 acetylation, PKM2 promotes radioiodine resistance in other ways. The therapeutic mechanism of radioactive ^131^I involves inducing DNA double-strand breaks via its emitted beta particles to kill cancer cells. However, cancer cells often repair these damages by activating DNA damage response pathways, leading to treatment resistance [128].

Meanwhile, RAI treatment causes cellular damage through the production of free radicals and ROS. To counteract this oxidative stress, cancer cells typically enhance their antioxidant defense systems. PKM2 can activate glucose-6-phosphate dehydrogenase (G6PD), a key enzyme in the pentose phosphate pathway (PPP), thereby increasing levels of NADPH and glutathione (GSH) [129]. NADPH and GSH are important reducing agents that can scavenge ROS generated by ^131^I therapy, protecting cancer cells from oxidative stress-induced death. This enhanced antioxidant capacity further promotes TC cells’ resistance to RAI.

Last but not least, PKM2 can form an axis with factors like HIF-1α, inducing the expression of EMT (epithelial–mesenchymal transition)-related genes, such as ZEB1, and stemness-related genes, such as OCT4 and NANOG, thereby promoting tumor cell dedifferentiation, migration, invasion, and cancer stem cell-like properties [130,131]. These characteristics are very likely to contribute to the development of radioiodine resistance. Nuclear PKM2, acting as an RNA-binding protein, specifically binds to G-quadruplex structures in pre-mRNA and prevents the binding of inhibitory RNA-binding proteins, thereby promoting the expression of rG4me (pre-mRNAs containing G-quadruplexes), a process also associated with EMT and tumor metastasis [105].

## 6. Therapeutic Strategies Targeting PKM2

**Figure 6:**
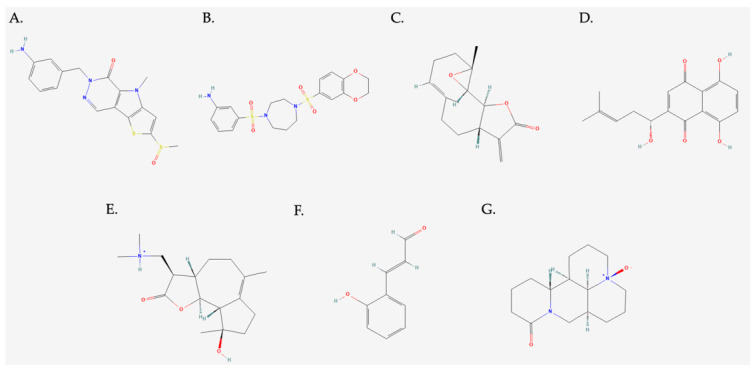
Chemical structures of the seven compounds evaluated in this study. All chemical structures were retrieved from the PubChem database (https://pubchem.ncbi.nlm.nih.gov) (accessed on 17 May 2026). (**A**) TEPP-46, (**B**) DASA-58, (**C**) Parthenolide, (**D**) Shikonin, (**E**) DMAMCL, (**F**) 2-Hydroxycinnamaldehyde, (**G**) Oxymatrine.

Based on the irreplaceable role of PKM2 in TC metabolic reprogramming, malignant progression, and radioiodine resistance, targeting PKM2 has become a potential direction for the precise treatment. According to the functional characteristics of PKM2, single-drug therapeutic strategies such as activating PKM2, inhibiting PKM2, and targeting upstream regulatory factors have been developed. Concurrently, based on its synergistic relationship with driver genes and the immune microenvironment, combined therapeutic strategies have shown better application prospects. The goal of the PKM2 activation strategy is to induce PKM2 to transform from the low-activity dimer to the high-activity tetramer through small-molecule compounds and stabilize its enzyme activity [32]. To systematically review the current progress in PKM2-targeted drug development and its status in thyroid cancer research, Table 2 categorizes and summarizes reported PKM2-targeted drugs, particularly highlighting those that have not yet been definitively validated in thyroid cancer. The chemical structures of TEPP-46, DASA-58, parthenolide, shikonin, DMAMCL, 2-hydroxycinnamaldehyde, and oxymatrine are shown in Figure 6. At present, representative small-molecule activators such as TEPP-46 and DASA-58 have been found, and their research in other cancer models provides experimental support for this strategy.

Research on TEPP-46 in kidney disease has made great progress. After TEPP-46 activates PKM2, it can inhibit the accumulation of HIF-1α, which has been proven to induce abnormal glycolysis and EMT in kidney cells. Inhibition of the EMT process can reduce the generation of fibroblasts and the deposition of extracellular matrix in the renal interstitium, thereby blocking the progression of renal fibrosis [132]. TEPP-46-mediated PKM2 activation can repair mitochondrial function, balance the metabolic imbalance between glycolysis and mitochondrial OXPHOS, reduce kidney cell damage, and delay the progression of diabetic kidney disease [133].

DASA-58 binds to the allosteric site of PKM2, prompting it to form a stable tetramer structure. This conformation is resistant to the inhibitory effect of tyrosine phosphorylated proteins, thereby continuously activating the pyruvate kinase activity of PKM2, reducing its nuclear translocation, and further regulating downstream metabolic and signaling pathways. DASA-58 can block LPS-induced HIF-1α expression in bone marrow-derived macrophages, downregulate HIF-1α-dependent target genes such as IL-1β, and inhibit inflammation-related metabolic reprogramming; enhance the metabolic switch from glycolysis to OXPHOS, reduce the aerobic glycolysis level of tumor cells, decrease lactate production and ATP supply, and inhibit tumor proliferation and metastasis; reduce PKM2 nuclear translocation, inhibit its binding to transcription factors (such as HIF-1α, β-catenin), and downregulate the expression of proliferation and invasion-related genes (such as GLUT1, HK2, VEGF) [134,135,136].

As a naturally derived PKM2 inhibitor, Shikonin can specifically bind to the active center of PKM2, inhibit its catalytic activity, and prevent the interaction between PKM2 and transcription factors, dual-blocking its enzymatic and non-enzymatic activities [137]. In vitro experiments have confirmed that Shikonin can significantly inhibit the proliferation of various subtypes of TC cells (papillary cancer, anaplastic cancer, medullary cancer), induce cell cycle arrest in the G2/M phase, activate the caspase-dependent apoptotic pathway, and has a more significant inhibitory effect on cancer cells with high PKM2 expression [138]. In BRAF V600E-positive TC cells, Shikonin can effectively block the BRAF-PKM2 crosstalk loop, downregulate the expression of pro-cancer genes such as GLUT1 and Cyclin D1, thus inhibiting cell invasion and metastasis [139].

**Table 2:** Current PKM2-targeting therapies.

Category	Agent(s)	Mechanism	Evidence in Thyroid Cancer?	References
Activators	TEPP-46 (PubChem CID: 44246499), DASA-58 (PubChem CID: 44543605)	Stabilizes PKM2 tetramer, enhances enzymatic activity, promotes pyruvate production, inhibits LPS-induced HIF-1α and IL-1β, attenuates proinflammatory M1 macrophages and promotes anti-inflammatory M2 macrophages.	No	[132]
	PA-12	Stimulates PKM2 pyruvate kinase activity (in vitro AC50 4.92 μM), inhibits lung cancer cell viability under hypoxia.	No	[140]
	Parthenolide dimers (PubChem CID: 6473881)	Activates PKM2 (AC50 15 μM), promotes tetramer formation, reduces nuclear translocation, inhibits proliferation and metastasis, induces apoptosis in glioblastoma cells.	No	[141]
Inhibitors	Shikonin (PubChem CID: 479503)	Direct PKM2 enzymatic inhibition, suppresses aerobic glycolysis, induces apoptosis, inhibits tumor growth.	No	[142]
	Naphthoquinone derivatives	Potent PKM2 inhibitors, induce apoptosis and autophagy through Akt/mTOR suppression.	No	[143]
	DMAMCL (PubChem CID: 133081974)	Targets PKM2, rewires aerobic glycolysis, suppresses glioblastoma cell proliferation and tumor growth.	No	[144]
	FV-429 (PubChem SID: 433986466)	Regulates nuclear translocation of PKM2, induces mitochondrial apoptosis, inhibits glycolysis in pancreatic cancer cells.	No	[145]
	2-Hydroxycinnamaldehyde (PubChem CID: 5318169)	Directly targets and inhibits PKM2, inhibits cancer cell proliferation and tumor growth.	No	[146]
	Oxymatrine (PubChem CID: 24864132)	Attenuates PKM2-mediated aerobic glycolysis, inhibits colorectal cancer metastasis.	No	[147]

## 7. Discussion and Future Directions

This review systematically summarizes the central role, regulatory network, specific functions, and targeted therapeutic strategies of PKM2 in TC metabolic reprogramming and occurrence and development. Combined with evidence from clinical samples, cell, and animal experiments, we establish PKM2’s intrinsic status as an indispensable pro-cancer molecule in TC.

Through its dual functions of enzymatic and non-enzymatic activities, PKM2 constructs a complex regulatory network in TC cells. On the one hand, in its low-activity dimeric form, it triggers the Warburg effect, shunting glycolytic intermediates to support tumor biosynthesis, thereby meeting the demands of rapid tumor cell proliferation for macromolecules, such as nucleotides, amino acids, and lipids. This metabolic reprogramming not only provides the necessary building blocks for tumor growth but also helps tumor cells adapt to various stresses within the TME, such as nutrient deprivation, hypoxia, and oxidative stress. On the other hand, PKM2 can translocate into the cell nucleus, acting as a transcriptional coactivator that synergizes with factors such as HIF-1α, β-catenin, and STAT3 to regulate the expression of genes related to cell proliferation, invasion, metastasis, and apoptosis resistance.

Concurrently, PKM2 forms specific regulatory axes with driver genes, like BRAF V600E, RAS, and c-Myc, which not only promote TC subtype progression and enhance malignant phenotypes, but also facilitate radioiodine resistance through the “BRAF-PKM2-NIS” axis, becoming a pivotal obstacle in the clinical diagnosis and treatment of TC. Furthermore, PTMs of PKM2 critically influence its conformation, stability, subcellular localization, and function, thereby finely tuning its enzymatic and non-metabolic roles. Hypoxia within the TME and M2 polarization of tumor-associated macrophages (TAMs) also regulate PKM2 expression and activity through mechanisms such as the HIF-1α pathway and cytokine secretion, forming a vicious cycle that drives tumor progression.

Based on PKM2’s fundamental role in TC, strategies targeting PKM2, including PKM2 activators, inhibitors, and indirect modulators, have shown potential application value. These strategies aim to inhibit tumor growth by stabilizing the high-activity tetrameric form of PKM2 or by blocking its enzymatic activity and non-metabolic functions. These studies provide novel insights and targets for the precise treatment of TC.

Despite significant progress in understanding PKM2’s role in TC, several research gaps remain, necessitating further exploration. Future research should focus on the following five dimensions:

i. Subtype-specific mechanism research

Significant differences exist in the biological characteristics, driver gene profiles, and metabolic patterns among various TC subtypes (differentiated, anaplastic, and medullary cancer). However, the subtype-specificity of the underlying PKM2 regulatory network remains unclear. Existing studies predominantly focus on PTC, with insufficient exploration of PKM2’s regulatory mechanisms in FTC, ATC and MTC. Moving forward, targeted research should be conducted for different subtypes to clarify variations in PKM2 expression levels, regulatory factors, and functions among various subtypes. For instance, elucidating the possible regulatory mode of the RET-RAS-PKM2 axis in MTC and the dominant role of PKM2 non-enzymatic activity in ATC will provide a theoretical basis for formulating subtype-specific targeted therapeutic strategies, avoiding “one-size-fits-all” treatment plans, and improving treatment accuracy [10,39].

ii. Exploration of TME interaction mechanism

TME is an integral factor affecting TC progression and treatment sensitivity [148]. At present, research on the role of PKM2 in the interaction between TC cells and microenvironment components is still scarce. Future work needs to focus on exploring the interaction mechanism between PKM2-mediated metabolic reprogramming and CAFs, immune cells. On one hand, it is crucial to clarify how metabolic products such as lactate and ROS, regulated by PKM2, regulate CAF activation and immune cell infiltration and function [109,149]. On the other hand, analyzing the reverse regulatory effect of cytokines secreted by microenvironmental components on PKM2 expression and activity will elucidate the vicious cycle mechanism of metabolic reprogramming and immune microenvironment remodeling, and provide more robust theoretical support for the combined application of PKM2-targeted drugs and immunotherapy [112,150].

iii. Optimization and innovation of targeted drugs

Existing PKM2-targeted drugs have problems such as poor tissue specificity, high off-target toxicity risk, and insufficient precision of function regulation, which restrict their clinical translation [151]. Future drug optimization should focus on two directions: First, developing thyroid tissue-specific delivery systems, such as nanocarriers coupled with thyroid-stimulating hormone receptors and NIS, to achieve precise delivery of PKM2 targeted drugs to TC cells, improve drug concentration in tumor tissues, and reduce damage to normal metabolically active tissues [104]; second, develop highly specific PKM2 allosteric modulators that break the “all-or-nothing” regulatory mode of existing drugs, and precisely regulate the enzymatic/non-enzymatic activities of PKM2 [151]. For example, for patients with TC radioiodine resistance, developing modulators that can selectively activate PKM2 enzymatic activity and restore NIS function without affecting its normal physiological functions would improve treatment safety and effectiveness [152].

iv. Clinical transformation verification of biomarkers

Existing evidence suggests that PKM2 has potential as a biomarker for TC, but it lacks verification in large-sample clinical cohort studies, and its clinical application value is not yet clear [121,153]. In further investigations, multi-center, large-sample clinical studies should be carried out to verify the biomarker function of PKM2 from three dimensions: First, its early diagnostic value, by detecting PKM2 expression level in serum and fine-needle aspiration samples, and clarifying its diagnostic efficacy for distinguishing benign and malignant thyroid lesions; Second, its prognostic evaluation value, by verifying the correlation between PKM2 expression level and patient DFS and OS through long-term follow-up, establishing its status as an independent risk factor for poor prognosis; Third, its efficacy prediction value, by focusing on analyzing the correlation between PKM2 expression and the efficacy of RAI therapy, BRAF inhibitor therapy, and PKM2 targeted therapy, constructing an efficacy prediction model based on PKM2, and realizing precise stratification and individualized treatment of patients [121,152].

v. Multi-omics integration to analyze regulatory networks

The role of PKM2 in TC is not regulated by a single pathway but involves a complex network of multiple levels, including metabolism, signaling pathways, and gene transcription [38,154]. In subsequent studies, it is necessary to carry out integrated analysis with the help of multi-omics technologies, such as transcriptomics, proteomics, metabolomics, and phosphoproteomics [119,124]. This approach will allow researchers to screen targeting genes and proteins regulated by PKM2 through transcriptomics and proteomics, clarify PKM2-mediated glycolytic flux changes and key metabolites through metabolomics, and construct a molecular network map of PKM2 regulation in TC through combined modeling of multi-omics data. This will help explore key targets that synergize with PKM2, provide new ideas for developing multi-target combined therapeutic strategies, and further improve the overall efficacy of TC treatment [155].

## 8. Conclusions

Despite significant progress in understanding PKM2’s role in thyroid cancer, numerous challenges and unresolved questions remain. Future research should focus on elucidating the precise regulatory mechanisms of PKM2 across different thyroid cancer subtypes, thoroughly exploring the complex interactions between PKM2 and the TME, and optimizing the specificity and efficacy of targeted drugs. Concurrently, large-scale clinical validation of PKM2 as a biomarker, coupled with multi-omics integration to analyze its regulatory network, will facilitate the translation of PKM2 from basic research to clinical practice. Ultimately, this will provide more precise and effective diagnostic and therapeutic strategies for thyroid cancer patients, significantly improving their prognosis and quality of life.

## Figures and Tables

**Table 1:** PKM2 regulation mechanisms in other tumors.

Regulatory Mechanism	Mechanism of Action	Effect on PKM2	Tissue	References
miR-122, miR-148a, miR-326	Binds to the 3′UTR of PKM2 mRNA	Inhibits PKM2 transcription	Hepatocellular carcinoma (HCC) and thyroid Cancer	[54,55]
Phosphorylation	FGFR1, AKT, ERK can phosphorylate at the Tyr105, Ser37, Thr365 sites, respectively. TRIM35 can inhibit phosphorylation at Tyr105.	Destabilize the active tetrameric PKM2 and lead to nuclear translocation of PKM2	FGFR1: Thyroid cancer AKT and ERK: No specific tumor TRIM35: HCC	[56,57,58,59,60]
Crotonylation	Crotonylation site at Lys305		Vascular smooth muscle cell	[61]
Succinylation	Succinylation at Lys498. The process can be reversed by SIRT5.		No specific tumor	[62,63]
O-GlcNAcylation	Targets Thr405 and Ser406, residues of the region.		No specific tumor	[64]
Acetylation	p300 can acetylate at Lys305 and Lys433 site of PKM2, while SIRT1 can reverse the acetylation at Lys305.		Breast cancer and non-small cell lung cancer (NSCLC)	[65,66,67,68,69]
Oxidative Modification	Oxidation at Cys358		No specific tumor	[70,71]
Metabolite Regulation				
Fructose-1,6-bisphosphate (FBP)	Binds to the allosteric site of PKM2	Induces its transformation from low-activity dimer to high-activity tetramer, significantly enhancing its glycolytic enzyme activity, inhibits nuclear translocation	No specific tumor	[72,73,74,75,76,77]
Serine	Competitively binds to the active center of PKM2	Competitively inhibits its enzyme activity, promotes nuclear translocation	No specific tumor	[78,79]

## Data Availability

No new data were created or analyzed in this study.

## Abbreviations

The following abbreviations are used in this manuscript:

PKM2	Pyruvate kinase muscle type 2
IARC	International Agency for Research on Cancer
DTC	Differentiated thyroid cancer
ATC	Anaplastic thyroid cancer
MTC	Medullary thyroid cancer
PTC	Papillary thyroid cancer
FTC	Follicular thyroid cancer
RAI	Radioactive iodine
RAI-R	RAI-refractory
MKIs	Multi-kinase inhibitors
ORR	Objective response rate
TME	Tumor microenvironment
ROS	Reactive oxygen species
PK	Pyruvate Kinase
PEP	Phosphoenolpyruvate
PKL	Pyruvate kinase liver type
PKR	Pyruvate kinase red blood cell type
PKM1	Pyruvate kinase muscle type 1
3′UTR	3′-untranslated region
PTM	Post-translational modification
O-GlcNAc	O-linked β-N-acetylglucosamine
TRIM35	Tripartite motif-containing protein 35
NSCLC	Non-small cell lung cancer
FBP	Fructose-1,6-bisphosphate
TKT	Transketolase
TCF	T-cell factor
LEF	Lymphoid enhancer factor
EMT	Epithelial–mesenchymal transition
OXPHOS	Oxidative phosphorylation
NIS	Na^+^-iodine symporter
TAMs	Tumor-associated macrophages
CAFs	Cancer associated fibroblast
DFS	Disease-free survival
OS	Overall survival
